# Intense pulsed light for inactivating planktonic and biofilm molds in food

**DOI:** 10.3389/fmicb.2022.1104875

**Published:** 2023-01-04

**Authors:** Xuejie Li, Nixuan Gu, Yanrui Ye, Haifeng Lan, Fang Peng, Gongyong Peng

**Affiliations:** ^1^School of Food Science and Engineering, Guangdong Province Key Laboratory for Green Processing of Natural Products and Product Safety, Engineering Research Center of Starch and Vegetable Protein Processing Ministry of Education, South China University of Technology, Guangzhou, China; ^2^Research Institute for Food Nutrition and Human Health, Guangzhou, China; ^3^School of Biology and Biological Engineering, South China University of Technology, Guangzhou, China; ^4^Department of Orthopeadic Surgery, The Third Affiliated Hospital of Guangzhou Medical University, Guangzhou, Guangdong, China; ^5^Department of Critical Care Medicine, The Third Affiliated Hospital of Guangzhou Medical University, Guangzhou, Guangdong, China; ^6^State Key Laboratory of Respiratory Diseases, National Clinical Research Center for Respiratory Diseases, National Center for Respiratory Medicine, Guangzhou Institute of Respiratory Health, The First Affiliated Hospital of Guangzhou Medical University, Guangzhou, Guangdong, China

**Keywords:** intense pulsed light, inactivation, planktonic, biofilm, *Aspergillus niger*, *Penicillium glaucum*

## Abstract

It has been reported that about a quarter of the world’s agriculture products is unable to be consumed each year because of mold contamination, resulting in incalculable economic losses. Despite modern food technology and the various preservation techniques available, the problem of mold contamination of food is still not adequately controlled. In this study, we simulated the biofilm formed by *Aspergillus niger* and *Penicillium glaucum* in liquid and solid food in 96 well cell culture plates and polycarbonate membrane models, respectively, and investigated the fungicidal effect of IPL on planktonic and biofilm molds at three different capacitance parameters at room and refrigerator temperatures. The results show that IPL can achieve fungicidal rates of over 99% for planktonic molds and over 90% for biofilm molds, and that the smaller the capacitance, the more frequent the irradiation required to achieve the same fungicidal rate. In addition, temperature, *A. niger* or *Penicillium glaucum* have no effect on the fungicidal effect of IPL. We believe that IPL is a promising non-thermal physical sterilization technique for fungal inhibition on food surfaces.

## 1. Introduction

Food spoilage caused by molds leads to enormous economic losses both in developing and developed countries. It is estimated that microbial contamination causes the loss of about 25% of the world’s agriculture products (Huis In’t Veld, 1996; Dantigny et al., 2005; Xu et al., 2012). Mycotoxins are metabolic substances produced by molds that are primarily toxic, and hazardous to human and animal health if inhaled, ingested, or even absorbed through skin contact (Liu J. et al., 2021). As of today, there are approximately 400 mycotoxins, and there are three main genera of mycotoxin producing molds associated with the human food chain, these are *Aspergillus*, *Penicillium*, and *Fusarium*. For example, the commonly occurring mycotoxins include aflatoxins produced by *Aspergillus flavus* and *Aspergillus parasiticus*, ochratoxin A produced by *Aspergillus carbonarius*, and fusarium toxins produced by *Fusarium* spp. (IARC, 1993). Molds can grow in a variety of different foods, including vegetables, fruit (Ferranti et al., 2018), meat, maize (Lee and Ryu, 2015), rice (Martín Castaño et al., 2017), coffee (Frisvad et al., 2004; Noonim et al., 2008), and nuts (Palumbo et al., 2015), due to their extreme adaptability to the environment.

Most of molds are disseminated through spores which can survive in extreme conditions, such as extremely high or low temperature, low oxygen concentration, high carbon dioxide concentration, low water availability, high osmotic pressure, and a wide range of pH (Dijksterhuis, 2017). The pigment in the spore walls is normally dark which may act as light shield protecting spores from UV damage (Wyatt et al., 2013). Spores are dispersed into the environment and will start growing again under suitable environmental conditions. Forming spores is an essential survival strategy for molds. Another strategy of the molds to resist extreme environments is the formation of biofilms. Biofilms generally consisting of exopolysaccharides, proteins, and nucleic acids, can strongly adheres to abiotic or biotic surfaces (Lin et al., 2017; Liu et al., 2022a,c). Biofilms are more resistant to antibiotics and biocidal agents (Xu et al., 2011; Miao et al., 2019; Li et al., 2020a). Therefore, molds in food are not easily killed. Food spoilage is an economic problem which, although there are modern food technologies and a wide range of available preservation techniques, is still not adequately controlled (Xu et al., 2019, 2021).

To avoid the molds spoilage of fruits, spraying with chemical fungicides including benomyl, thiabendazole, and imazalil is a common practice. However, chemical fungicides have a high potential to cause health and environmental issues because of chemical residues (Misra et al., 2019). Ethanol, generally regarded as safe (GRAS) in the United States, has long been used as a mold inhibitor to control fruit and food products decay. Small residues on the surface of food after ethanol treatment may affect the taste and quality of food (Gabler et al., 2005; Dao and Dantigny, 2011). It also reported that some essential oils extracted from several plants, such as thyme, cinnamon, clove, and oregano, are also considered as potential sources to control the growth of *Aspergillus* and *Penicillium* in food (de Carvalho et al., 2015). UV light is normally used for inactive microorganisms including *A. flavus* and *P. corylophilum* in various places, but the disinfection effect of UV radiation against fungal spores is limited to the part of the object that can be irradiated by UV light (Begum et al., 2009). The inactivation of molds by cold plasma is a disinfection method of high interest to the food industry because it requires low energy input and has a milder effect on quality (Misra et al., 2019; Liu et al., 2022b).

In this study, we are going to introduce another effective method to sterilize mold named intense pulsed light (IPL), which is a non-thermal processing technology. The IPL device consists of a control module which acts as a power supply and a treatment chamber which consists of three components xenon lamp, intense pulsed light, and a shelf (Hwang et al., 2015; Liu Z. et al., 2021). The xenon lamp produces a spectrum of 200–1,100 nm for short-time, high power, and broad-spectrum radiation to inactivate molds on the target surface. The mechanism of IPL sterilization is that the UV, visible, and infrared rays in IPL act synergistically on microorganisms, destroying their genetic material DNA and RNA, effectively killing pathogenic microorganisms, and inhibiting the reproduction of bacteria and viruses for a certain period of time (Babilas et al., 2010). It has been reported that IPL was used to inactivate foodborn gram-positive bacteria (Liu Z. et al., 2021), *Pseudomonas aeruginosa* (Yi et al., 2017), *Listeria monocytogenes* (Cheigh et al., 2013), *Escherichia coli* O157: H7 (Cheigh et al., 2012; Li et al., 2020b), and *Cronobacter sakazakii* (Chen et al., 2020). But there is no report on the use of IPL for fungicide. So, this study focuses on the inactivation efficiency of IPL to inactivate the planktonic cells and biofilms of two molds most likely to cause food spoilage, *Aspergillus niger* and *Penicillium glaucum*.

## 2. Materials and methods

### 2.1. Strains used in this study

*Aspergillus niger* BM-ANI-1 and *P. glaucum* BM-PGL-1 were originally given to us as a gift by another group who isolated the two strains themselves and were stored in our laboratory at −80°C in 60% glycerol.

### 2.2. Strains activation and amplification culture

*Aspergillus niger* and *P. glaucum* were preserved on solid PDA (Huankai, Guangzhou, China) medium and stored at-20°C. A single colony was picked into 2 ml of liquid YPD medium and incubated overnight in a shaker at 37°C and at 200 rpm. 100 μl of overnight culture was transferred to 3 ml of fresh YPD medium and incubated in a shaker at 37°C for 4 h at 200 rpm. The strains are most active at this point and can be used for subsequent experiments.

### 2.3. Planktonic molds cultures

To ensure the fungi form single colonies on PDA plates, the fungal solution obtained from 2.2 was diluted to 10^5^ CFU/ml. Then transferred 100 μl of the diluted bacterial solution onto a PDA agar plate and spread well. Waited until the surface of the agar plate was dry and then irradiated with intense pulsed light.

### 2.4. Biofilm formation in 96-well cell culture plate model

The fungal solution obtained from 2.2 was diluted to a final concentration of 10^5^ CFU/ml. The diluted fungal solution was then transferred to a 96 well cell plate with 200 μl per well. The 96 well cell plates were incubated in a 37°C incubator for 8 h (early biofilm) and 2 days (mature biofilm), with fresh medium changed every 24 h. The suspension was gently removed from 96 well cell plates after incubation and washed three times with 200 μl of sterile saline to remove planktonic molds. Then, the 96 well cell plates were placed in the IPL chamber, located 15 cm directly below the pulsed lamp for irradiation. Subsequently, the plate was scraped by adding 200 μl of saline to each well, and the scraping was repeated three times. A total of 600 μl of the suspension collected was placed in a 2 ml centrifuge tube and mixed on a mixer at the highest rate for 2 min, followed by a dilution plate count method to detect their culturable number.

### 2.5. Biofilm formation in polycarbonate membrane model

The polycarbonate membrane was sterilized by placing both sides under a UV lamp for 20 min and repeating twice to ensure that the polycarbonate membrane was completely sterile. The polycarbonate membrane was placed smooth side up in the middle of a solid plate, and 5 μl of the final concentration of 10^5^ CFU/ml of fungal solution was dropped onto the polycarbonate membrane. The solid plates were incubated in a 37°C incubator for 8 h (early biofilm) and 2 days (mature biofilm), with fresh medium changed every 24 h. After incubation, the plates were placed in the IPL chamber, located 15 cm directly below the pulsed lamp for irradiation. Then the colonized membrane was transferred with the polycarbonate membrane to a shaking tube containing 5 ml of sterile saline, and the colonized membrane was dispersed and detached from the polycarbonate membrane using an ultrasonic crusher. The instrument parameters were 50%, 125 W, 20 kHz, sonication for 5 s, stopping for 5 s, and cycling three times. The sonicated biofilm solution was mixed for 2 min at the highest rate using a mixer, followed by a dilution plate count to detect the culturable number.

### 2.6. Counting method of live molds

100 μl of fungal solution was taken in the first well of the eight-linked tube and 180 μl of sterile saline was added to each well, starting with the second well. Subsequently, 20 μl was removed from one well to the second well, blown and stirred with a displacement gun, and then 20 μl was removed from the second well to the third well, and so on. In this way, each well corresponds to a concentration of 10^0^, 10^−1^, 10^−2^, 10^−3^… Eight gradient drops of each sample were taken for counting, 10 μl at a time, with three parallel settings.

### 2.7. Intense pulsed light treatment

For the planktonic fungi, the number of irradiations was 15, 30, and 45 for a capacitance of 650 μF, 30, 45, and 60 for a capacitance of 470 μF, and 60, 90, and 120 for a capacitance of 220 μF, with a blank control for each capacitance condition. For 96 well cell culture plate model, the number of irradiations was 60, 180, and 360 for a capacitance of 650 μF, 180, 360, and 540 for a capacitance of 470 μF, and 360, 540, and 720 for a capacitance of 220 μF, with a blank control for each capacitance condition. For polycarbonate membrane model, the number of irradiations was 180, 360, and 540 for a capacitance of 650 μF, 540, 720, and 900 for a capacitance of 470 μF, and 900, 1,080 and 1,260 for a capacitance of 220 μF, with a blank control for each capacitance condition. The fungicidal effect of IPL was strain specific and the number of irradiations was adjusted according to the fungicidal rate.

To detect the fungicidal effect of IPL at normal temperature (25°C) and low temperature (4°C), the IPL device was put at room temperature (approximately 25°C) and in a refrigerator (4°C), respectively, when it was in operation.

### 2.8. Statistical analysis

When the experimental group was compared with the control group, One-way ANOVA was used to determine if the difference was statistically significant. 0.01 < *p* value < 0.05 (marked as *), 0.001 < *p* value < 0.01 (marked as **), and value of *p* < 0.001 (marked as ***) were set as statistically different, statistically significantly different, and extremely statistically significantly different, respectively.

## 3. Results

### 3.1. The fungicidal effect of IPL on planktonic *Aspergillus niger* and *Penicillium glaucum*

**Figure 1:**
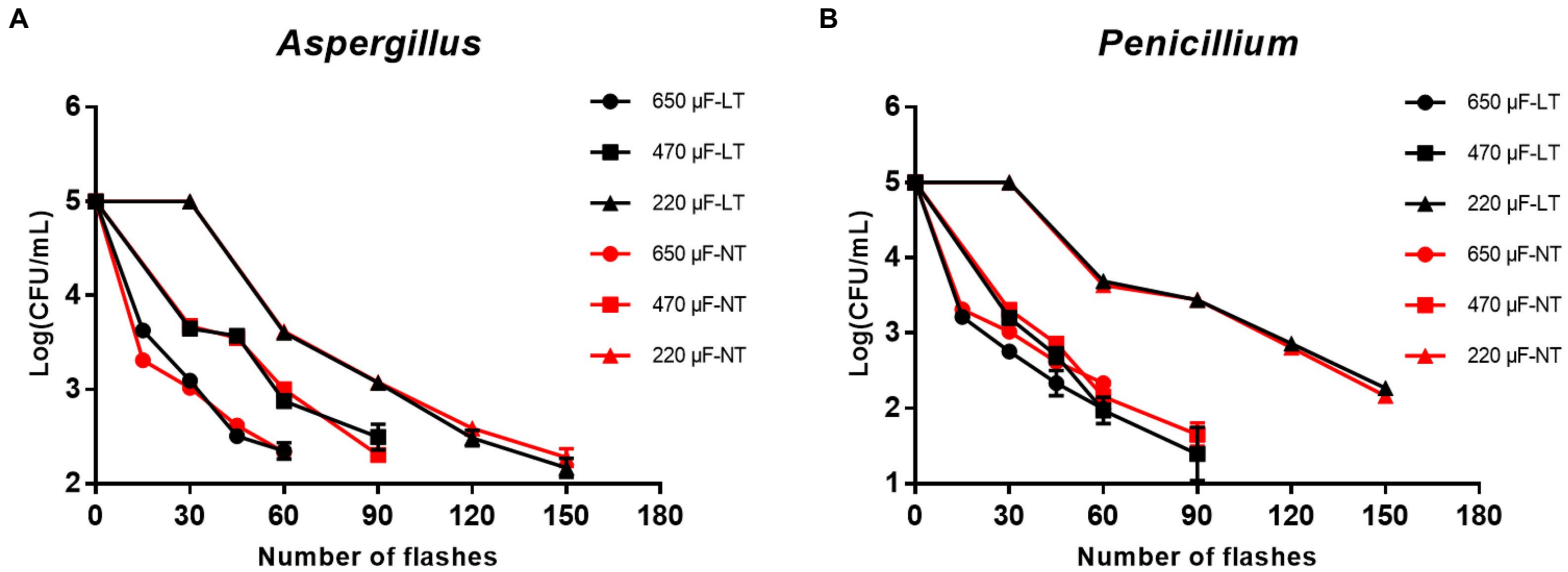
Reduction in cultivable number of *Aspergillus niger* and *Penicillium glaucum* in planktonic cultures. **(A)** Reduction in cultivable number of *A. niger*. **(B)** Reduction in cultivable number of *P. glaucum* [650, 470, and 220 μF are three different capacitances of IPL. LT and NT mean low temperature (4°C) and normal temperature (25°C), respectively].

First of all, to investigate the inactivation rate of IPL on two molds in their planktonic state, plates obtained from 2.3 planktonic molds cultures were treated with three different capacitances and the number of irradiations. The results show that to achieve >99% sterilization of *A. niger* at room temperature, the IPL parameters need to be set to 45 irradiations with a capacitance of 650 μF, 60 irradiations with a capacitance of 470 μF, and 120 irradiations with a capacitance of 220 μF. The IPL parameters required to achieve >99% sterilization of *A. niger* in a refrigerated environment (4°C) are the same as those required under normal temperature conditions (Figure 1A; Table 1). The IPL parameters required to achieve >99% lethality against *P. glaucum* at room temperature and refrigerated conditions are the same as, they are 45 irradiations with a capacitance of 650 μF, 45 irradiations with a capacitance of 470 μF, and 120 irradiations with a capacitance of 220 μF (Figure 1B; Table 1). Both at room temperature and refrigerator temperature, IPL is able to sterilize *A. niger* and *P. glaucum* by more than 95% for three different capacitances corresponding to three different irradiation times, respectively. To achieve the same effect of killing the molds, the smaller the capacitance, the greater the number of the irradiations required (Figure 1; Table 1). Also, from the data in the Figure 1, it can be seen that the IPL parameters required to achieve the same fungicidal rate for *A. niger* and *P. glaucum* in planktonic state are essentially the same.

**Table 1:** Inactivation rate of IPL on planktonic cultures.

Temperature	Strain	Capacitance					
		650 μF		470 μF		220 μF	
		The number of irradiations	Rate of inactivation	The number of irradiations	Rate of inactivation	The number of irradiations	Rate of inactivation
RT	*Aspergillus*	15	95.12%	30	95.26%	60	95.86%
		30	98.58%	45	96.46%	90	98.80%
		45	99.49%	60	99.00%	120	99.61%
	*Penicillium*	15	97.94%	30	97.95%	60	95.69%
		30	98.95%	45	99.27%	90	97.22%
		45	99.58%	60	99.86%	120	99.70%
4°C	*Aspergillus*	15	95.74%	30	95.55%	60	95.96%
		30	98.75%	45	96.28%	90	98.82%
		45	99.68%	60	99.24%	120	99.69%
	*Penicillium*	15	98.35%	30	98.41%	60	95.09%
		30	99.42%	45	99.49%	90	97.24%
		45	99.77%	60	99.90%	120	99.47%

### 3.2. The fungicidal effect of IPL on *Aspergillus niger* and *Penicillium glaucum* in the 96 well cell culture plate model

**Figure 2:**
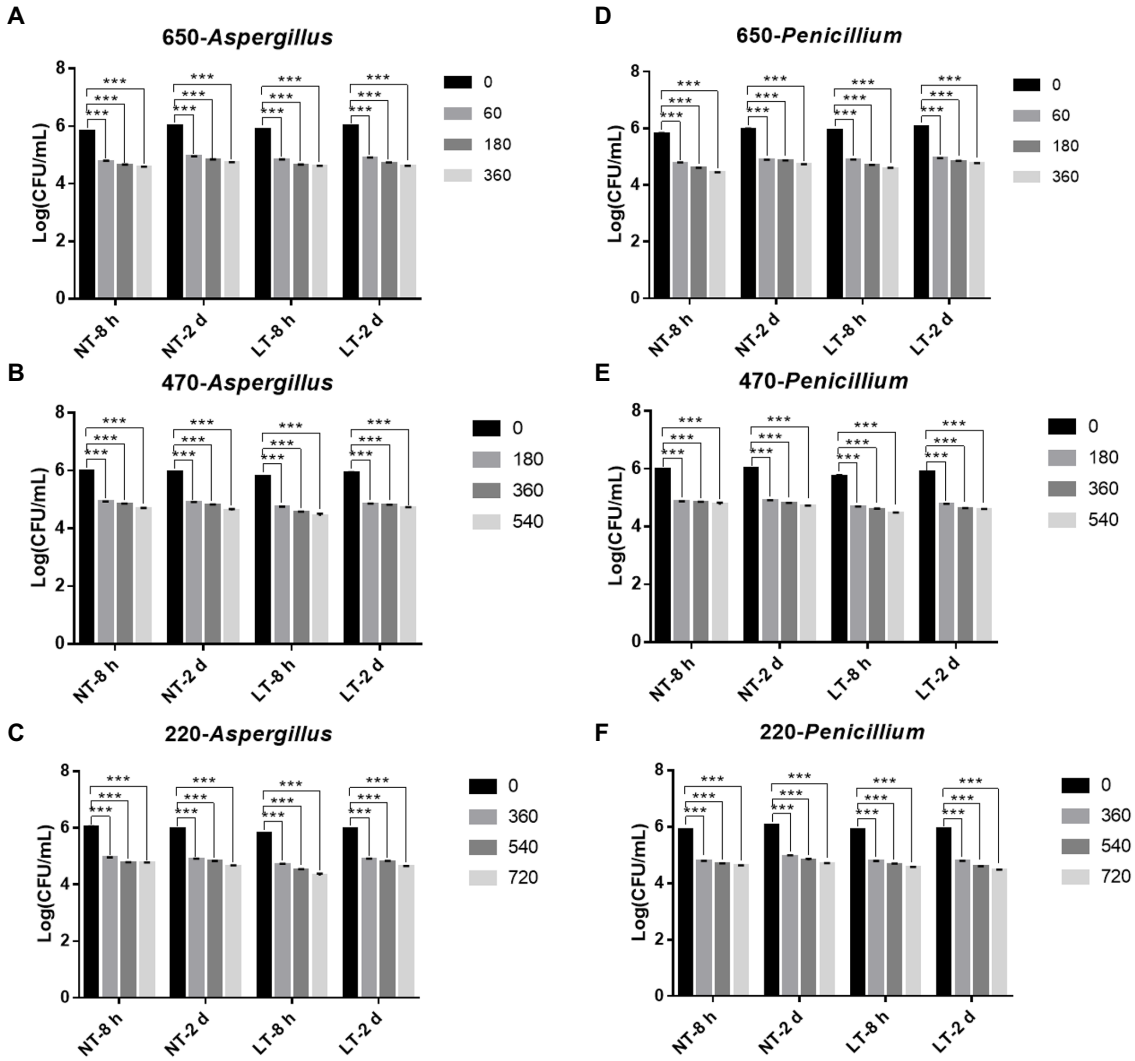
IPL sterilization efficiency on biofilm of *Aspergillus niger* and *Penicillium glaucum* under different capacitances in the 96 well cell culture plate model. **(A–C)** IPL sterilization efficiency on biofilm of *A. niger*. **(D–F)** IPL sterilization efficiency on biofilm of *P. glaucum*. (650 μF: 0.0407 J*cm^−2^, 470 μF: 0.0319 J*cm^−2^, and 220 μF: 0.0150 J*cm^−2^). The asterisks denote statistical significance as determined by One-way ANOVA test (^***^p < 0.001, ^**^0.001 < *p* < 0.01, ^*^0.01 < *p* < 0.05). Error bars indicate SD from three independent experiments.

Then, to study the fungicidal effect of IPL on biofilm in a liquid environment, we simulated the biofilm formed in a 96 well cell culture plate as a biofilm in a liquid environment. And to investigate the relationship between the fungicidal effect of IPL and the maturity of the biofilm, two different states of biofilm, early biofilm (8 h) and mature biofilm (2 days; Liu Z. et al., 2021), were used for the test. As can be seen in Figure 2, for early biofilm and mature biofilm of *A. niger* and *P. glaucum*, IPL at three different capacitance parameters resulted in a highly significant reduction in the number of molds compared to the control group (*p* < 0.001). Specifically, IPL can achieve over 90% fungicidal activity against *A. niger* and *P. glaucum*, and approximately 95% fungicidal activity was achieved at 360 irradiations at 650 μF, 540 irradiations at 470 μF, and 720 irradiations at 220 μF.

Consistent with the planktonic state, there was no significant difference in the fungicidal effect of IPL on *A. niger* and *P. glaucum* biofilms formed on 96-well plate. However, *A. niger* and *P. glaucum* require a higher number of irradiations to kill in biofilm with the same capacitance compared to *A. niger* and *P. glaucum* in the planktonic state, but the maturity of the biofilms has no effect on the fungicidal effect. Moreover, there was no significant change in the fungicidal effect of IPL at room temperature and refrigerator temperature (Figure 2; Supplementary Table S1).

### 3.3. The fungicidal effect of IPL on *Aspergillus niger* and *Penicillium glaucum* in the polycarbonate membrane model plate

**Figure 3:**
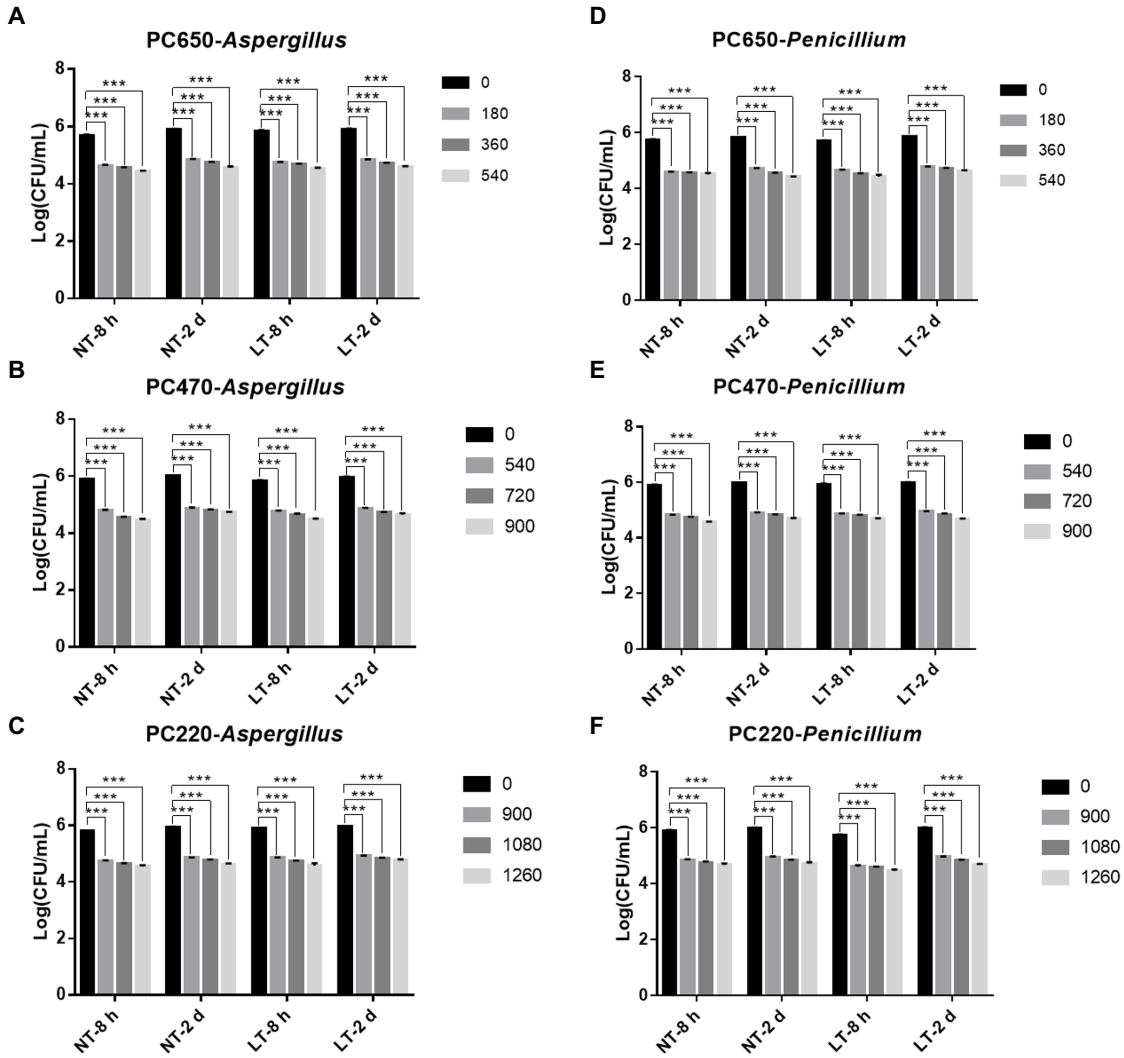
IPL sterilization efficiency on biofilm of *Aspergillus niger* and *Penicillium glaucum* under different capacitances in polycarbonate membrane model. **(A–C)** IPL sterilization efficiency on biofilm of *A. niger*. **(D–F)** IPL sterilization efficiency on biofilm of *P. glaucum*. (650 μF: 0.0407 J*cm^−2^, 470 μF: 0.0319 J*cm^−2^, and 220 μF: 0.0150 J*cm^−2^). The asterisks denote statistical significance as determined by One-way ANOVA test (^***^p < 0.001, ^**^0.001 < *p* < 0.01, ^*^0.01 < *p* < 0.05). Error bars indicate SD from three independent experiments.

To study the fungicidal effect of IPL on biofilm in a solid environment, we simulated the biofilm formed in the polycarbonate membrane model plate as a biofilm in a solid environment. Same to the model in the 96-well plate, two different states of biofilm, early biofilm (8 h) and mature biofilm (2 days), were used for the test. As can be seen in Figure 3, for early biofilm and mature biofilm of *A. niger* and *P. glaucum* in the polycarbonate membrane model plate, IPL at three different capacitance parameters resulted in a highly significant reduction in the number of molds compared to the control group (*p* < 0.001). Specifically, IPL can achieve over 90% fungicidal activity against *A. niger* and *P. glaucum*, and approximately 95% fungicidal activity was achieved at 540 irradiations at 650 μF, 900 irradiations at 470 μF and 1,260 irradiations at 220 μF.

There was no significant difference in the fungicidal effect of IPL on *A. niger* and *P. glaucum* biofilms formed in the polycarbonate membrane model. However, *A. niger* and *P. glaucum* require a higher number of irradiations to kill biofilm in the polycarbonate membrane model plate with the same capacitance compared to *A. niger* and *P. glaucum* in the 96 well cell culture model, but the maturity of the biofilms has no effect on the fungicidal effect (Figure 3; Supplementary Table S2). From the above three experimental results, it is clear that *A. niger* and *P. glaucum* in the planktonic state are the most easily killed, followed by biofilm in 96 well cell culture plate, and finally biofilm in the polycarbonate membrane model. Moreover, there was also no significant change in the fungicidal effect of IPL at room temperature and refrigerator temperature.

## 4. Discussion

For *A. niger* and *P. glaucum*, the IPL capacitance of 650, 470, and 220 μF at a given number of flashes achieves a sterilization rate of over 90%, and the higher the capacitance, the lower the number of flashes required to achieve the target rate. No significant difference in the sterilization effect of IPL on the two molds at room temperature (25°C, NT) and refrigerated (4°C, LT) temperature. And the maturity of the biofilm does not affect the effectiveness of IPL sterilization. But it is clear that planktonic molds used in this study are more easily killed than molds in biofilms, which is in line with our expectations. Molds can accumulate in biofilms which can serve to protect microorganisms. Once embedded in this matrix, microorganisms tend to become resistant to the action of disinfectants, antibiotics, and UV light (Dosti et al., 2005).

Compared to planktonic bacteria, such as *Staphylococcus aureus* ATCC25923, *Listeria monocytogenes* ATCC19118, and *Bacillus cereus* ATCC14579 (Xu et al., 2007; Li et al., 2020a; Liu Z. et al., 2021), planktonic molds require more frequent irradiation to be killed at the same capacitance. We speculate that there are several reasons for the difference in the fungicidal effect of IPL on fungi and bacteria. Bacteria do not have a nucleus surrounded by a nuclear membrane, while molds have a nucleus formed by a nuclear membrane; Bacteria are organisms made up of a single cell, and molds are made up of multiple cells; prokaryotic cells are generally smaller, typically 1–10 μm in diameter, while eukaryotic cells are larger, typically 10–100 μm in diameter; the composition of the cell wall differs: the main component of the bacterial cell wall is peptidoglycan, whereas the main component of the fungal cell wall is chitin.

The environment in which microorganisms thrive can also affect the sterilization effect of IPL. Hee-Jeong Hwang and colleagues found that the bactericidal effect of IPL varies considerably even in different liquid samples, such as in mineral water, carbonated drinks, and coffee, because of the differences in absorption properties and light transparency (Hwang et al., 2015). The most important factor in determining the effectiveness of IPL inactivation is the beam incident on the sample, in addition to factors, such as product area, thickness, transparency, color, viscosity, presence of particulate matter, type of microorganism, and absorption characteristics of the food (Salehi, 2022). One of the shortcomings of this study is that it does not show the bactericidal effect of IPL on bacteria on food, data on which we will subsequently publish in a separate paper.

As a fast, safe, energy-saving and environmentally friendly non-thermal physical sterilization method, IPL has a good sterilization effect on common food-borne spoilage microorganisms and pathogenic microorganisms, providing a theoretical basis for the application of IPL to food surface sterilization. At the same time, the low-temperature environment of refrigeration has little effect on the sterilization effect of IPL, providing the possibility of applying IPL sterilization technology to low-temperature food storage cabinets such as refrigerators and cold stores, in order to extend the storage date of food.

## 5. Conclusion

This study describes that IPL can achieve over 90% fungicidal rates against planktonic and biofilm *A. niger* and *P. glaucum* at room and refrigerator temperatures. The lower the capacitance the more irradiation is required to achieve the same fungicidal effect. For the same capacitance, biofilm *A. niger* and *P. glaucum* require more irradiation than planktonic to be killed. The biofilm in the polycarbonate membrane model plate is more difficult to kill than the biofilm in the 96 well cell culture plates. Further studies including the use of IPL for sterilization during food processing and sterilization during food preservation in the refrigerator are needed to illustrate the sterilization rate of IPL against molds in food. Also, testing the effect of using IPL on the taste, flavor, and appearance of food is needed.

## Data availability statement

The original contributions presented in the study are included in the article/Supplementary material, further inquiries can be directed to the corresponding authors.

## Author contributions

XL: writing—original draft and data curation. NG: resources and conceptualization. YY: methodology and supervision. HL: writing—review and editing. FP: methodology and conceptualization. GP: supervision and review and editing. All authors contributed to the article and approved the submitted version.

## Funding

This work was supported by Natural Science Foundation of Guangdong (2021A1515011024), Guangdong-Hong Kong-Macao Joint Laboratory of Respiratory Infectious Disease (GHMJLRID-Z-202118), and the Independent Project of State Key Laboratory of Respiratory Disease (SKLRD-Z-202103), 111 Project (B17018).

## Conflict of interest

The authors declare that the research was conducted in the absence of any commercial or financial relationships that could be construed as a potential conflict of interest.

## Publisher’s note

All claims expressed in this article are solely those of the authors and do not necessarily represent those of their affiliated organizations, or those of the publisher, the editors and the reviewers. Any product that may be evaluated in this article, or claim that may be made by its manufacturer, is not guaranteed or endorsed by the publisher.

## Supplementary material

The Supplementary material for this article can be found online at: https://www.frontiersin.org/articles/10.3389/fmicb.2022.1104875/full#supplementary-material
