# Supplementary material for: Intense pulsed light for inactivating planktonic and biofilm molds in food
Source: Front Microbiol. 2023 Jan 4;13:1104875. doi: 10.3389/fmicb.2022.1104875 (PMC9846768; doi:10.3389/fmicb.2022.1104875)
Supplement: Supplementary file 1 [file Table_1.docx]

**Supplementary data**

Table S1. Inactivation rate of IPL on biofilm in the 96 well cell culture plate model.

Table S2. Inactivation rate of IPL on biofilm in the polycarbonate membrane model.
